# Plant Small RNA World Growing Bigger: tRNA-Derived Fragments, Longstanding Players in Regulatory Processes

**DOI:** 10.3389/fmolb.2021.638911

**Published:** 2021-06-07

**Authors:** Cristiane S. Alves, Fabio T. S. Nogueira

**Affiliations:** ^1^ Cold Spring Harbor Laboratory, Cold Spring Harbor, NY, United States; ^2^ Laboratório de Genética Molecular do Desenvolvimento Vegetal, Departamento de Ciências Biológicas, ESALQ/USP, Piracicaba, Brazil

**Keywords:** tRNAs, translation regulation, tRFs, signaling, stress response

## Abstract

In the past 2 decades, the discovery of a new class of small RNAs, known as tRNA-derived fragments (tRFs), shed light on a new layer of regulation implicated in many biological processes. tRFs originate from mature tRNAs and are classified according to the tRNA regions that they derive from, namely 3′tRF, 5′tRF, and tRF-halves. Additionally, another tRF subgroup deriving from tRNA precursors has been reported, the 3′U tRFs. tRF length ranges from 17 to 26 nt for the 3′and 5′tRFs, and from 30 to 40 nt for tRF-halves. tRF biogenesis is still not yet elucidated, although there is strong evidence that Dicer (and DICER-LIKE) proteins, as well as other RNases such as Angiogenin in mammal and RNS proteins family in plants, are responsible for processing specific tRFs. In plants, the abundance of those molecules varies among tissues, developmental stages, and environmental conditions. More recently, several studies have contributed to elucidate the role that these intriguing molecules may play in all organisms. Among the recent discoveries, tRFs were found to be involved in distinctive regulatory layers, such as transcription and translation regulation, RNA degradation, ribosome biogenesis, stress response, regulatory signaling in plant nodulation, and genome protection against transposable elements. Although tRF biology is still poorly understood, the field has blossomed in the past few years, and this review summarizes the most recent developments in the tRF field in plants.

## Introduction

Transfer RNA (tRNA) is an ancient non-coding RNA molecule whose canonical role is to bridge the information contained in messenger RNAs (mRNAs) to protein synthesis (Giegé, 2008). Furthermore, tRNAs were likely part of an RNA genome replication mechanism in the so-called “RNA world,” where before the advent of protein synthesis, plants might have retained tRNA-like motifs in their genomic RNA, which evolved as 3′terminal structures that tagged RNA genome for replication (Maizels and Weiner, 1994; Giegé, 2008). Not only are tRNAs indispensable for the translational machinery, but these molecules also play non-canonical roles, for example in apoptosis inhibition (Mei et al., 2010), breast cancer metastasis, neuronal homeostasis regulation (reviewed by Schimmel, 2018), as stress sensors and gene regulators through uncharged tRNAs, as well as being used as primers for reverse transcription by viruses (Phizicky and Hopper, 2010). In the past few decades, significant advances in high-throughput sequencing technologies have enabled the discovery of new classes of small RNAs in plants. Among them, tRNA-derived fragments (tRFs) arise as new enigmatic molecular players.

When fragments of tRNAs were first observed, they were believed to be degradation byproducts. However, an increasing number of reports have led to a change in this view. Indeed, the accumulation of tRFs in the cell is unlikely a restricted byproduct of tRNA degradation since tRNAs that did not pass quality control are adenylated as a signal for degradation (Chernyakov et al., 2008; Copela et al., 2008). Furthermore, tRNAs are cleaved at different positions to generate tRNA fragments, giving rise to four possible classes according to the cleavage position. Short fragments that range from 17 to 26 nucleotides (nt) are classified into three categories: 1) 5′tRF: cleavage at the D-loop at the 5′ end of a mature tRNA; 2) 3′tRF: cleavage at the T-loop at the 3′end, including CCA; 3) 3′U-tRF: cleavage at the 3′trailer of the tRNA precursor. The last category, 5′ and 3′tRNA-halves, are cleaved at the anticodon loop and include longer fragments, ranging from 30 to 40 nucleotides. The latter class notably includes the tRNA-derived stress-induced RNAs (tiRNAs), which correspond to the tRNA-halves induced by stress—a nomenclature commonly used for mammals. In addition to these categories, internal tRNA fragments (i-tRFs) that range from 19 to 36 nt, were detected only in humans (Telonis et al., 2015; Karousi et al., 2020). Although there is still no consensus in the nomenclature, here the 5′tRF, 3′tRF, 3′U-tRF, and 5′or 3′tRNA-halves nomenclature will be used.

Transfer RNA-derived fragments gained increasing interest when Lee and Collins demonstrated that during amino acid starvation, *T. thermophila* mature tRNAs were cleaved at the anti-codon loop, generating tRNA-halves in coordination with the cell cycle progression (Lee and Collins, 2005). Several pieces of evidence suggest that the accumulation of tRNA-halves is evolutionarily conserved and that it could be part of a protein synthesis regulatory pathway that responds to stress and fluctuates during the life cycle (Haiser et al., 2008; Jöchl et al., 2008; Thompson et al., 2008; Pederson, 2010; Wang et al., 2016). The smaller classes of tRNA-derived fragments also rose as potential regulatory molecules. Like tRNA-halves (or tiRNAs), smaller tRFs are also associated with stress response, and they might participate in the RNA interference (RNAi) machinery or even be integrated into a new RNAi-like pathway. However, there is still controversy in the field, as tRFs processing by DICER (or DICER-LIKE) proteins as well as tRFs loading into ARGONAUTES are not clear (Loss-Morais et al., 2013; Kumar et al., 2014; Alves et al., 2017; Cognat et al., 2017; Martinez et al., 2017; Megel et al., 2018). This review focuses on the new findings on plant tRNA-derived fragments and their implications.

## Transfer RNA Fragments Biogenesis

The mechanisms responsible for processing tRNA-derived fragments are still poorly understood, especially in plants. For instance, the mechanism by which 3′U-tRFs are processed in plant cells is likely to be similar to 3′U-tRFs cleaved by the RNase Z in human cells, but it remains to be experimentally confirmed (Lee et al., 2009; Haussecker et al., 2010).

In mammalian cells, tRF-halves can be processed by Angiogenin (ANG), a member of the RNase A family. ANG is primarily localized in the nucleus, however, it can also be found in the cytoplasm, where it is associated with RNH1 (RNase H inhibitor 1). RNH1 inhibits ANG activity in normal conditions but releases ANG under stress conditions (Yamasaki et al., 2009; Li and Hu, 2012). Rny1p (RNase in Yeast 1), a member of RNase T2 family in yeast, and the non-specific single-stranded RNA nuclease T2/S superfamily RNS (comprising S-LIKE RIBONUCLEASES 1–5) in plants can process 5′tRFs and 3′tRFs, as well as tRNA-halves (Thompson and Parker, 2009; Alves et al., 2017; Megel et al., 2018). In plants, *S-LIKE RIBONUCLEASE 1* (*RNS1*) is upregulated during phosphate starvation (Bariola et al., 1994) and is tightly regulated in response to the phytohormone Abscisic Acid (ABA) and to wounding stress (Hillwig et al., 2008). Arabidopsis RNS1 is responsible for the production of both 5′tRFs (Ala) and 5′tRNA-halves (Ala and Asp) (Megel et al., 2018). Furthermore, the expression of T2 RNases is triggered by stress responses, coinciding with the high-level accumulation of most tRNA-halves. Interestingly, Arabidopsis *RNS1* is expressed in specific tissues and *RNS1* may be responsible for processing tRF-halves in specific cell types and developmental stages (Nowacka et al., 2013; Alves et al., 2017; Megel et al., 2018).

The major players in small RNA processing are the RNases III Dicer, Drosha, and, in plants, DICER-LIKE (DCLs). DCL activity requires double-stranded RNA (dsRNA), which can originate from hairpin structures or by the synthesis of dsRNA from single-stranded RNA (Gasciolli et al., 2005; Henderson et al., 2006). Some specific 5′tRFs and 3′tRFs in mammals are reported to be Dicer-dependent (Babiarz et al., 2008; Cole et al., 2009; Haussecker et al., 2010). In plants, DCLs are unlikely to play a constitutive role in tRF processing (Alves et al., 2017; Megel et al., 2018). However, DCLs may be involved in tRF processing during specific developmental stages or in specific tissues. For instance, it was shown that DCL1 is responsible for the cleavage of specific 19-nt tRFs in Arabidopsis pollen (Martinez et al., 2017). Interestingly, 19-nt 5′tRFs-accumulate at higher levels in pollen in Arabidopsis, maize, and rice — as well as in *Physcomitrella patens* gametophore and sporophyte — than in other tissues, suggesting specific processing of tRFs in plant male gametes (Martinez et al., 2017).

## Transfer RNA Fragments and Germ Cells: Keeping Retrotransposons in Check

Although transposable elements (TEs) presence and movement influence genome structure and dynamics, their activity is likely to disrupt genome stability, thus requiring rigorous control (Slotkin and Martienssen, 2007; Lisch, 2013). The RNAi machinery is the main factor in most eukaryotes that protects the genome from naturally active, or stress-activated, TEs by using TE-derived small RNAs and piRNAs to degrade TEs transcripts (Slotkin and Martienssen, 2007; Slotkin et al., 2009; Martinez et al., 2016; Ramat and Simonelig, 2021). Recently, tRFs have been reported to be part of the initial trigger for regulatory pathways occurring at specific retrotransposons in both plants and mammals (Martinez et al., 2017; Schorn et al., 2017).

Retrotransposons, retroviruses, and pararetroviruses contain in their primer binding site (PBS) a region that is targeted by tRNAs, mainly by the 3′extremity of certain tRNAs that are used as a primer for retrotranscription (Marquet et al., 1995). In cancer cell lines, a subset of 22-nt 3′CCA-tRFs halves have a complementary sequence to retrotransposons such as LINEs (Long Interspersed Nuclear Elements) and LTRs (Long Terminal Repeats), and these 22-nt 3′CCA-tRFs might interfere with RNA expression by inducing the RNAi pathway (Kawaji et al., 2008). In mouse embryonic stem cells, epigenetic reprogramming takes place, and LTRs are expressed due to changes in the chromatin conformation, whereas 18-nt 3′tRFs accumulate and block reverse transcription of those elements by targeting the PBS, the same mechanism also seems to take place during viral infection (Schorn et al., 2017; Schorn and Martienssen, 2018). Furthermore, 5′tRFs may also have the ability to regulate LTRs in mammal’s embryonic stem cells and embryos (Sharma et al., 2016; Boskovic et al., 2020).

**FIGURE 1:**
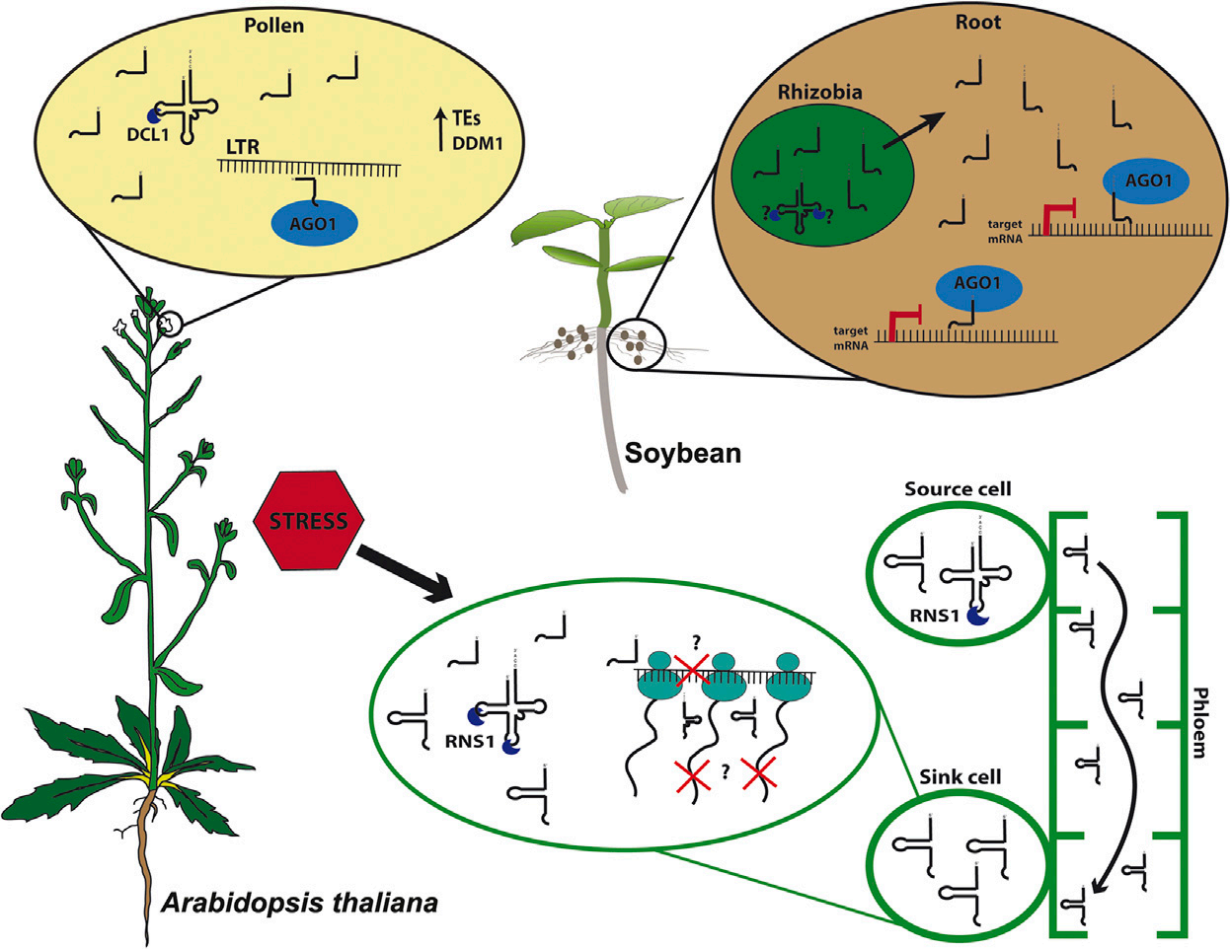
tRNA-derived fragments (tRFs) biogenesis and function. In *Arabidopsis thaliana* pollen grains, 5′tRFs accumulate due to the expression of *DECREASE IN DNA METHYLATION 1* (*DDM1*) and are processed by DICER-LIKE1 (DCL1) and loaded into ARGONAUTE1 (AGO1), targeting Long Terminal Repeat (LTR) transposable elements (TEs). Under stress conditions, plants as Arabidopsis triggers tRFs and tRNA-halves processing intermediated by S-LIKE RIBONUCLEASE 1 (RNS1). 5′tRFs can modulate translation, and the D-loop structure likely plays role in the efficiency of translation inhibition. Meanwhile, tRNA-halves act as signaling molecules traveling through the phloem-sap from source cells toward sink cells where they accumulate and may disrupt translation. In soybean (*Glycine max*), rhizobial tRFs processing is still unknown. However, they are found in symbiotic roots where they are loaded into AGO1, targeting transcripts responsible for root development, playing an important role in the symbiotic regulation between bacteria and root.

In Arabidopsis, 5′tRFs accumulate in mature pollen grains, a tissue where TEs are reactivated due to the loss of heterochromatin during epigenetic reprogramming, a mechanism characteristic of germinative cells (Figure 1; Slotkin et al., 2009; Martinez et al., 2017). A good model for studying TE activation and its consequences on plant development is the Arabidopsis mutant *ddm1*, lacking the chromatin remodeler DECREASE IN DNA METHYLATION 1 (DDM1), which shows global loss of heterochromatin and hence TEs reactivation (Slotkin et al., 2009). Interestingly, *ddm1* accumulates tRFs not only in mature pollen grains but also in other tissues such as inflorescences. The expression of some tRNA genes is likely influenced by the loss of *DDM1*, which could partly explain the tRF accumulation in different tissues in this mutant. Interestingly, the double mutant *ddm1;dcl1* loses tRFs in pollen, suggesting a microRNA-like pathway in this specific tissue, supporting the hypothesis of a tissue-specific tRF biogenesis pathway (Martinez, 2017). Interestingly, Arabidopsis microRNAs miR845 and miR1511 also target LTRs and are evolutionarily linked to tRNA iMet^CAT^ (Šurbanovski et al., 2016), which produces a 19-nt 5′tRF that targets the *Gypsy* element *Athila6A* (Martinez et al., 2017). Together, these observations suggest a complex mechanism of plant TE regulation, which employs distinct small RNA classes.

## Processing Transfer RNA Fragments: Aiding Stress Response

The accumulation of tRFs and tRNA-halves in specific tissues and at specific developmental stages is likely tightly regulated, however, this regulation is still not well understood (Hsieh et al., 2009; Zhang et al., 2009; Alves et al., 2017; Cognat et al., 2017). Nevertheless, it is well established that different stresses can trigger tRNA cleavage to produce tRFs/tRNA-halves. Fragments from tRNAs were first observed during amino acid starvation in *T. thermophila* (Lee and Collins, 2005). Reports from Arabidopsis showed tRNA-halves, 3′tRFs, and 5′tRFs accumulation during oxidative stress, indicating a potentially conserved yet unknown mechanism to cope with stress (Thompson et al., 2008; Alves et al., 2017).

Plant microRNA accumulation is affected by glucose stress, a key regulator of developmental processes (Duarte et al., 2013). In contrast, Arabidopsis tRFs do not seem to respond to mannitol or glucose stress, suggesting that tRFs might not play a direct role in growth and development triggered by sugar signaling (Alves et al., 2017). However, tRFs were shown to be involved in the response to abiotic stresses such as drought, salt, cold, and heat (Loss-Morais et al., 2013; Wang et al., 2016; Alves et al., 2017; Byeon et al., 2017; Byeon et al., 2019). Distinct species respond differently to the same environmental changes, indicating a mechanism with specific variables depending on the species. For example, the Arabidopsis 19-nt 5′tRF-Arg^CCT^ is upregulated in response to drought stress while, in rice, the same tRF, does not differentially accumulate under drought stress but is upregulated under cold stress (Loss-Morais et al., 2013; Alves et al., 2017). Similarly, the wheat tRF-Tyr^GUA^ accumulates under heat, drought, salt, and heat stress (Wang et al., 2016). In *Brassica rapa*, heat stress for a short time triggers a reduction of 5′tRF-Glu transcripts and an increase in 5′tRF-Asp transcripts in leaves, ovules, embryo, and endosperm (Byeon et al., 2017). Interestingly, the progeny of stressed *B. rapa* plants also exhibited differential levels of tRFs: a decrease in 5′tRF-Ala, tRF-Arg, and tRF-Tyr, and a higher increase in tRF-Asp (Byeon et al., 2019).

Plastid tRFs were also reported in Arabidopsis, where specific mitochondrial and chloroplastic tRFs were detected and accumulate outside the organelles, making 1 and 25%, respectively, of the total tRF content. Moreover, 5′tRFs originated from these organelles were enriched in AGO1 immunoprecipitation and accumulate during cold stress (Cognat et al., 2017). Similar to Arabidopsis, the generation of tRNA-halves and tRFs was reported to be modulated by abiotic stresses (i.e., heat stress) in *B. rapa* chloroplasts (Wang et al., 2011). The correlation between stress response and changes in tRF accumulation is becoming clearer. Nevertheless, more work needed be done to unravel the mechanism(s) by which tRFs participate in distinct plant stress response pathways (Figure 1).

Not only environmental stresses, but nutrients and phytohormones can influence the production of tRFs. Barley tRFs undergo changes in abundance under the presence and absence of phosphorous (Hackenberg et al., 2013), while phosphate deficiency in Arabidopsis leads to the production of 19-nt 5′tRF-Asp^GTC^ and 5′tRF-Gly^TCC^ in roots, but not in shoots (Hsieh et al., 2009). ABA treatment of tomato leaves leads to an overall decrease in the accumulation of tRFs, mainly the 20-nt 5′tRF-Ala, suggesting the implication of this specific tRF in ABA response (Luan et al., 2020).

## Signaling Molecules and Translation Modulators

Despite the complexity of tRF biology and of the mechanisms involved in spatio-temporal and stress responses that they appear to be involved in, deciphering the function of tRFs has improved over the past few years. The first clue came from pumpkin, where tRNA-halves, among other small non-coding RNAs (ncRNAs), accumulate in the phloem sap, probably playing a role in translation inhibition mechanisms (Zhang et al., 2009). In higher plants, there are only two tRNA families that contain introns, tRNA-Mete (elongator Methionine) and tRNA-Tyr (Michaud et al., 2011). Interestingly, all tRNAs found in the pumpkin phloem sap belong to tRNA genes lacking introns, indicating that aberrant splicing is not the source of tRNA-halves production. Intriguingly, although *ex vivo* processing of tRNA-Met only occurs in leaves and stems, fragments of tRNA-Met (still unclear if these fragments derive from tRNA-Mete or Meti, initiator Methionine) were found in the phloem sap, suggesting that these tRNA-halves could be involved in long-distance signaling. It is possible that these tRNA-halves inhibit protein synthesis, as demonstrated when these tRNA-derived fragments were added to an *in vitro* translation system (Zhang et al., 2009). Moreover, in Arabidopsis, some specific tRFs are found in shoots, yet accumulate more in roots, despite the tRNA that these tRFs derive from are found at similar levels in both tissues. This observation, along with the findings that they accumulate in pumpkin phloem sap, suggest a possible conserved mechanism for long-distance movement (Figure 1; Hsieh et al., 2009; Hsieh et al., 2010).

Additional evidence supporting the role of tRFs in translational regulation was demonstrated in Arabidopsis. *In vitro* experiments have shown that several tRNA-derived fragments containing 5′-TOG-like (5′terminal oligoguanine), such as the tRNA-halves -Ala, -Leu, and -Cys (Nowacka et al., 2013) or 5′tRF-Ala^AGC^, 5′tRF-Asn^GUU^, are capable of inhibiting translation (Lalande et al., 2020). In mammals, the four guanines (G) residues at the 5′ end of the tRNA-half (Ala) enable G-quadruplex formation, a structure essential for translation repression (Ivanov et al., 2014). Although G-quadruplex displaces the eukaryotic initiation factor eIF4G/A in mammal mRNA, thus inhibiting the binding of the small ribosomal subunit (Lyons et al., 2020), this mechanism has not been shown in plants. In mammals, inhibiting translation relies on the ability to form a G-quadruplex structure, and the inhibition efficiency is correlated with the type of structure, presence of 5′TOG or 5′ secondary structure (Ivanov et al., 2014; Jackowiak et al., 2017). Although this might be true for mammalian cells, the same effect was not observed in Arabidopsis (Jackowiak et al., 2017). Recently, G-quadruplex structures were detected in Arabidopsis and rice, with different folding predictions in both species and developmental stages, suggesting that this structures could be spatio‐temporal specific (Yang et al., 2020). The 4 Gs present in Arabidopsis tRNA-half (Ala) are not essential to affect protein synthesis. On the other hand, 2 G residues—that belong to the conserved D-loop nucleotides in 5′tRF-Ala and 5′tRF-Asn—seem to be necessary, although the presence of the G residues alone is not sufficient to explain the specificity of the inhibition (Lalande et al., 2020). Despite the exciting possibility that some tRFs can interfere with translation, experiments are usually performed using synthetic tRNA fragments that lack the post-transcriptional modifications present in all tRNAs, likely interfering with binding affinity *in vivo*. Considering the amount and complexity of post-transcriptional modifications harbored by tRNAs, collecting post-transcriptionally modified tRFs from fractions might be the best approach to test the tRNA-fragment potential role in modulating transcription and translation (Lalande et al., 2020). Recent new genome-wide technologies, such as PANDORA-seq (panoramic RNA display by overcoming RNA modification aborted sequencing), allow the identification of previously undetected tRNA-derived small RNAs (Shi et al., 2021), and they may help to test novel roles of tRFs in translation. Alternatively, taking advantage of techniques developed to study microRNA functions, such as short tandem target mimic (STTM) (Tang et al., 2012) would avoid the tRNA modifications concerns as a consequence of introducing synthetic tRNA-fragments molecules.

Indeed, STTM was used to silence specifics tRFs, building evidence on the tRFs involved in cross-kingdom signaling between bacteria and plants. Rhizobia, a symbiotic, nitrogen-fixating bacteria in legume nodules, was shown to regulate soybean nodules formation using tRFs (Figure 1). The Rhizobia produces specific 21-nt tRFs, 3′tRFs-Val^CAC^, 3′tRF-Gly^UCC^, and 5′tRF-Gln^CUG^, that positively regulate soybean rhizobial infection and nodulation by repressing soybean genes involved in root and root hair development. Moreover, those tRFs might be loaded in AGO1, suggesting a mechanism similar to the canonical biogenesis and action of microRNAs (Ren et al., 2019).

Even though evidence of AGO proteins and tRFs network is growing, mechanisms and validation of its interaction are however not well established. Indeed, translation repression appears to be independent of mRNA sequence, suggesting an RNAi-independent pathway (Lalande et al., 2020). Furthermore, tRFs can associate with actively elongating polyribosomes, but not efficiently with ribosomal subunits, supporting the possible existence of an unknown yet conserved tRF-mediated translation regulation, even though the mechanisms by which tRFs associate with active polyribosomes are unknown (Lalande et al., 2020). In human cells, data suggest that 3′tRFs affect the 30S ribosomal subunit 5′ external transcribed spacer, reducing 18S rRNA levels (Kim et al., 2017). tRNA-halves are thought to interact directly with the ribosomal machinery, and it is therefore unlikely that these molecules are incorporated into AGOs and participate in the RNAi pathway (Martinez, 2017; Lalande et al., 2020). In contrast, 19-nt 5′tRFs in Arabidopsis inflorescence and pollen are loaded into AGO1 to target *Gypsy* (LTRs) elements. This observation is an indicative of a specialized function in germinative cells (Martinez et al., 2017) as discussed previously.

## Discussion and Conclusion

The biogenesis and mechanisms of plant tRNA-fragment are poorly understood. Recently, the field expanded and brought to light new and exciting knowledge. The RNS family is responsible for processing some tRFs and tRNA-halves (Alves et al., 2017; Megel et al., 2018), but DCL1 might play an important role in the male gamete-specific tRF biogenesis, where they likely help control TEs and may participate in a miRNA-like pathway (Martinez et al., 2017). Specific nuclear and plastid tRNA-halves, 5′ and 3′tRFs were demonstrated to be part of the stress response-associated pathways, although the mechanisms are unclear and vary among different plant species (Wang et al., 2011; Loss-Morais et al., 2013; Wang et al., 2016; Alves et al., 2017; Cognat et al., 2017; Byeon et al., 2017; Byeon et al., 2019). *In vitro* and *ex vivo* experiments are leading the way to demonstrate the potential role of tRNA-derived fragments in the control of transcription and translation (Hsieh et al., 2009; Zhang et al., 2009; Hsieh et al., 2010; Lalande et al., 2020), although data to uncover these mechanisms are yet to be demonstrated. Cross-kingdom transcription modulation was revealed between Rhizobia and soybean, where tRFs produced by the bacteria are capable of silencing specific host genes (Ren et al., 2019). Despite the lack of a mechanistic pathway, this evidence is important and demonstrate a potential application of tRFs in improving specific agronomic traits. AGO role in the tRF pathway is still to be determined (Martinez et al., 2017; Ren et al., 2019; Lalande et al., 2020), and reports should be addressed carefully to eliminate ambiguous conclusions. The confirmation that tRFs could function in a microRNA cannonical patway would potentially make it easier to understand the biological role of these RNA fragments due to the extensive knowledge in the microRNA field.

tRNA-derived fragments are intriguing molecules composing an additional layer of small RNA-mediated regulation conserved in several organisms. The evidence so far suggests that possible biogenesis mechanisms and biological roles go hand in hand. tRNA-halves and tRFs may require different RNases for processing, depending on the tRNA isotype, tissue, and species, that such specificity is required by their biological roles and, thus that there is no unified mechanism to describe this class of small RNAs. On the other hand, tRNAs are primordial molecules and, therefore, it is conceivable that tRFs were part of an ancient regulatory mechanism in the “RNA world,” their conserved and essential roles eventually being complemented or substituted by other pathways. Both tRNAs and their derived fragments are difficult to study due to their complex structures and numerous chemical modifications. Modern high-throughput sRNA sequencing technologies (such as PANDORA-seq) are slowly bypassing these caveats to generate reproducible large-scale data. Advances in direct RNA sequencing could help unravel the mechanistic secrets associated with these ancient and crucial molecules and piece together the mysteries of the tRNA-derived fragments.
